# Evocalcet improved the PTH–calcium setpoint and suppressed parathyroid proliferation in mice model of primary hyperparathyroidism

**DOI:** 10.1007/s00774-026-01699-y

**Published:** 2026-02-17

**Authors:** Yasuo Imanishi, Tomoe Hirakawa, Waka Haruyama, Keigo Tsushida, Mariko Sakai, Tetsuya Kitayama, Takehisa Kawata, Emi Donoue, Keisuke Inoue, Ikue Kobayashi, Yuki Nagata, Masafumi Kurajoh, Tetsuo Shoji, Andrew Arnold, Masanori Emoto

**Affiliations:** 1 Department of Vascular Medicine, Vascular Science Center for Translational Research, Osaka Metropolitan University Graduate School of Medicine, Osaka, Japan; 2 Department of Metabolism, Endocrinology and Molecular Medicine, Osaka Metropolitan University Graduate School of Medicine, Osaka, Japan; 3 Biomedical Science Research Laboratories 1, Research Unit, R&D Division, Kyowa Kirin Co., Ltd, Shizuoka, Japan; 4 Medical Affairs Department, Kyowa Kirin Co., Ltd., Tokyo, Japan; 5 Department of Research Support Platform, Osaka Metropolitan University Graduate School of Medicine, Osaka, Japan; 6 Center for Molecular Oncology and Division of Endocrinology and Metabolism, University of Connecticut School of Medicine, Farmington, CT USA

**Keywords:** Calcimimetics, Calcium-sensing receptor, Cinacalcet, Evocalcet, Parathyroid hormone, Primary hyperparathyroidism

## Abstract

**Background:**

Evocalcet is an allosteric modulator of the calcium-sensing receptor (CaSR) that effectively suppresses parathyroid hormone (PTH) secretion in both primary and secondary hyperparathyroidism. However, its effects on the PTH-calcium setpoint and parathyroid cell proliferation remain unclear.

**Methods:**

We investigated these effects using the PC mouse model of primary hyperparathyroidism, which is characterized by parathyroid-targeted cyclin D1 overexpression . Evocalcet was administered orally at a dose of 0.025 mg/g diet. The PTH-calcium setpoint was evaluated, and the antiproliferative effect of evocalcet on parathyroid cells was assessed using 5-bromo-2′-deoxyuridine (BrdU) incorporation assays. The effects of evocalcet were compared with those of cinacalcet. Expression levels of the vitamin D receptor (VDR) and CaSR in parathyroid glands were also examined.

**Results:**

Evocalcet significantly reduced the PTH-calcium setpoint in PC mice, restoring it to levels comparable to those observed in wild-type controls. Evocalcet treatment markedly decreased the proportion of BrdU-positive parathyroid cells, indicating suppression of parathyroid cell proliferation. This antiproliferative effect was comparable to that observed with cinacalcet. Neither evocalcet nor cinacalcet altered VDR or CaSR expression in the parathyroid glands.

**Conclusions:**

Evocalcet, similar to cinacalcet, lowers the PTH-calcium setpoint and inhibits parathyroid cell proliferation in a mouse model of primary hyperparathyroidism. These findings suggest that evocalcet may not only reduce PTH secretion but also attenuate disease progression in hyperparathyroidism.

## Introduction

Three types of receptors on parathyroid cells that contribute to calcium and phosphate homeostasis by controlling parathyroid hormone (PTH) secretion from parathyroid cells are calcium-sensing receptor (CaSR), vitamin D receptor (VDR), and fibroblast growth factor receptor (FGFR)-Klotho complex [1]. The regulation of PTH secretion by calcium is known to be altered in patients with primary hyperparathyroidism (PHPT) and secondary hyperparathyroidism of uremia (SHPT), as evidenced by changes in the PTH–calcium curve and the setpoint of calcium [2]. The setpoint, often defined as the calcium concentration required to reduce maximal PTH secretion by half, is an important indicator of parathyroid gland sensitivity to extracellular calcium [3]. The elevation of the PTH–calcium setpoint in PHPT and SHPT has been partially attributed to the reduction of CaSR expression on the surface of parathyroid cells [4]. This decrease in CaSR density may contribute to a desensitization of the parathyroid glands to extracellular calcium levels, resulting in abnormal regulation of PTH secretion.

Calcimimetics are a class of medications that modulate the activity of the CaSR, which is primarily located on the surface of parathyroid cells [5]. These agents are frequently utilized in clinical practice, especially in the management of conditions associated with abnormal PTH secretion. Currently, four types of calcimimetics, such as cinacalcet, etelcalcetide, evocalcet, and upacicalcet, are available for clinical use to suppress PTH secretion from parathyroid glands; however, evocalcet and upacicalcet are currently marketed exclusively in Japan.

Chronic kidney disease–mineral and bone disorder (CKD–MBD) is a serious health problem that causes severe adverse outcomes, including fracture, cardiovascular disease, and premature death not only in patients with chronic kidney disease. [6] SHPT is frequently observed in CKD–MBD patients on maintenance hemodialysis, which contributes to fracture, ectopic calcification, cardiovascular disease, and an increased risk of death [6]. Calcimimetics, such as cinacalcet [7], etelcalcetide [8] evocalcet [9], and upacicalcet [10], successfully decrease the serum PTH levels in patients with SHPT on hemodialysis.

PHPT, most frequently caused by sporadic parathyroid adenomas, is associated with morbidities such as fractures, kidney stones, CKD, and finally increased risk of death [11]. Only cinacalcet [12, 13] and evocalcet [14] are approved for the treatment of PHPT, whereas etelcalcetide and upacicalcet are not indicated for this condition. This is primarily because these agents have been developed and approved specifically for SHPT in CKD patients on dialysis. Their predominant renal elimination and dependence on hemodialysis for clearance make them less practical and potentially unsafe for use in non-CKD patients, where their pharmacokinetics have not been well studied.

Cinacalcet, a widely used CaSR agonist for hyperparathyroidism, has clinical limitations, including nausea, vomiting, and CYP2D6 inhibition; GI intolerability often limits the dose of cinacalcet and causes poor compliance or discontinuation [15, 16]. Through systematic optimization, evocalcet was developed as an oral allosteric CaSR agonist with enhanced activity and improved pharmacokinetics to offer a superior option for hyperparathyroidism [17]. While cinacalcet induced a significant delay in gastric emptying in rats, evocalcet caused no delay [18] due to less vagus nerve action potentials by evocalcet compared to cinacalcet [19] Head-to-head comparison of evocalcet with cinacalcet demonstrated the non-inferiority of evocalcet to cinacalcet in suppressing intact parathyroid hormone with fewer gastrointestinal-related adverse events in patients on maintenance hemodialysis [9]

As evocalcet was developed from cinacalcet, both compounds bind to the same amino acid, Glu837, within the 7-transmembrane regions of the CaSR [17]. Both evocalcet and cinacalcet exhibit positive allosteric effects on CaSR in vitro, with evocalcet being designed to mimic the effects of cinacalcet [18, 20]. However, evocalcet’s impact on the PTH–calcium setpoint and parathyroid cell proliferation in PHPT remains unclear. This study investigates the effect of evocalcet on the PTH–calcium sigmoidal curve and its inhibitory potential on parathyroid gland proliferation in a PHPT mouse model with parathyroid-targeted overexpression of the cyclin D1 oncogene [21].

## Materials and methods

### Materials

Evocalcet was synthesized by the Mitsubishi Tanabe Pharma Corporation.

### Animals

FVB/N background PTH-cyclin D1 transgenic mice, which exhibit parathyroid-targeted overexpression of the human *cyclin D1* oncogene, were used in this study as a model of primary hyperparathyroidism (PC) [21]. Parathyroid-targeted overexpression of the *cyclin D1* oncogene not only results in the development of abnormal parathyroid cell proliferation, but the mice also develop chronic biochemical hyperparathyroidism with an increased PTH–calcium setpoint [22]. All mice were provided with the commercially available rodent diet, CE-2 (Clea Japan, Inc., Tokyo, Japan), containing 1.14% Ca and 1.08% phosphate. Food and water were available ad libitum. Animal studies were approved by the Osaka Metropolitan University Graduate School of Medicine.

### Experimental protocols

WT mice were used for time-course changes after the oral injection of various doses of evocalcet (1, 3, 10, 30 mg/kg BW) suspended in 0.5% methylcellulose solution, and blood samples were collected at 0, 2, 6, 24, and 48 h after administration, to estimate an appropriate dose of evocalcet to administer to the mice. Similar analyses using cinacalcet HCl (cinacalcet) were performed previously [23]. In the following experiments, 0.025 mg evocalcet/g chow, 1 mg cinacalcet/g chow, or vehicle was administered.

### PTH–calcium setpoint analyses

The setpoint analyses were performed on 16 PC mice and 8 WT mice (64–77-week-old) (Table 1). The 16 PC mice were then randomly assigned into 2 equal groups for the analysis. Evocalcet (0.025 mg/g in chow) or vehicle was administered for 11 days, and the PTH–calcium setpoint analyses were performed from the third day to the 10th day after the start of the administration.

**Table 1:** Serum parameters and body weight of the mice for setpoint analyses in PC and WT mice before treatments

Subjects	WT vehicle (*N* = 8)	PC vehicle (*N* = 8)	PC evocalcet (*N* = 8)
Serum iPTH (pg/mL)	211 ± 96	495 ± 282^a^	356 ± 155
Serum calcium (mg/dL)	8.7 ± 0.2	10.8 ± 0.4^a^	10.7 ± 0.7^a^
Serum phosphate (mg/dL)	5.7 ± 0.5	4.7 ± 0.7^a^	4.4 ± 0.7^a^
Body weight (g)	44.4 ± 4.5	43.8 ± 4.2	37.6 ± 8.4

Data are presented as mean ± SD

^a^*P* < 0.05 versus WT vehicle by Tukey–Kramer

PTH–calcium setpoint analyses were performed using a previously described method [24]. Briefly, each mouse was administered a single intraperitoneal injection of the indicated dose of calcium gluconate or EGTA in saline. To change the serum calcium levels, mice were administered calcium gluconate (300 µmol/kg BW) or EGTA (300 and 450 µmol/kg BW), and blood was collected at 30, 60, and 90 min after the administration. When the lowest serum calcium was not below 7.0 mg/dL for untreated PC mice or 6.0 mg/dL for evocalcet-treated PC mice, 600 and 750 µmol/kg BW EGTA were administered.

To obtain the setpoint, the maximum and minimum PTH secretion levels, and the slope at the setpoint from the relationship between serum PTH concentration and serum calcium concentration (mediated by calcium gluconate and/or EGTA administration), sigmoidal curves were generated by the four-parameter models as previously described [3]. For setpoint analysis, PTH–calcium data from each mouse were fitted individually using a four-parameter logistic (sigmoidal) model [24]. Briefly, the PTH concentration was assigned to the response role, and the serum calcium concentration was assigned to the regressor role, and a sigmoidal curve was obtained using a logistic 4P model from each mouse using JMP software, version 12.0.1 (SAS Institute, Cary, NC). Setpoint data from each mouse were fitted individually to a four-parameter logistic model, yielding mouse-specific estimates of maximum PTH, minimum PTH, setpoint, and Hill slope. Goodness-of-fit was assessed for each individual model using AICc, BIC, SSE, MSE, RMSE, and R^2^.

### Parathyroid gland proliferation analyses

Parathyroid gland proliferation analyses were performed on 30 PC mice and 10 WT mice (59–71-week-old) as previously described [23]. The 30 PC mice were randomly assigned into 3 equal groups for the analysis. Evocalcet, cinacalcet, or vehicle was administered for 10 days and then sacrificed under medetomidine hydrochloride, midazolam, and butorphanol tartrate combined anesthesia, and both parathyroid glands were removed. All mice received 240 µg/day of subcutaneous 5-bromo-20-deoxyuridine (BrdU) continuously via Alzet micro-osmotic pump (model 1007D; DURECT Corporation, Cupertino, CA 95014), implanted subcutaneously for the last 5 days before sacrifice.

### Measurement of biochemical parameters

Blood samples were collected by tail nicking or cardiac puncture, and serum was collected immediately after coagulation of the blood. Serum was stored at − 80 °C until assayed for serum concentrations of calcium, phosphate, creatinine, and PTH. Serum calcium, phosphate, and creatinine levels were measured using diagnostic kits (FUJIFILM Wako Pure Chemical Corporation, Osaka, Japan). Serum PTH levels were measured using the mouse PTH enzyme immunoassay (Quidel Corporation, Athens, OH, USA).

### Parathyroid immunohistochemistry for CaSR and VDR

Resected parathyroid glands were immediately fixed in 4% paraformaldehyde for 24 h and embedded in paraffin. Tissue sections (3 μm) were cut and mounted on glass slides as previously described [23, 25]. Deparaffinized sections were treated with Peroxidazed 1 (Biocare Medical, Pacheco, CA, USA) for 5 min to block endogenous peroxidase activity. The antigen retrieval method, Heat-Induced Epitope Retrieval (HIER), was used. Sections were treated with HistoVT ONE (NACALAI TESQUE INC, Kyoto, Japan) at 90 °C for 1 min (CaSR) or 40 min (VDR).

After washing with TBS, the sections were incubated with blocking solution A (Histofine mouse stainkit; Nichirei Bioscience, Tokyo, Japan) for 60 min, followed by three washes in TBS for CaSR immunohistochemistry. Sections were then incubated with anti-CaSR mouse monoclonal antibody (Novus Biologicals, LLC, Centennial, CO, USA) (diluted 1:15,000) overnight at 4 °C in a humidified chamber. After washing with TBS, sections were incubated with blocking solution B for 10 min, and then incubated with HRP-conjugated anti-mouse antibody (Histofine Simple stain MAXPO; Nichirei Bioscience, Tokyo, Japan) as appropriate for 10 min at room temperature.

For VDR immunohistochemistry, sections were incubated with 5% Normal Goat Serum for 60 min. Sections were then incubated with anti-VDR Rabbit polyclonal antibody (Cell Signaling Technology, Massachusetts, USA) (diluted 1:1,000) overnight at 4 °C in a humidified chamber. After washing with TBS, sections were incubated with HRP-conjugated anti-rabbit antibody (EnVisiom + /HRP, Rabbit; Agilent, CA, US) for 30 min at room temperature.

For visualization of all immunoreactions, sections were stained using 3, 3’-diaminobenzidine DAB (Histofine DAB substrate kit; Nichirei Bioscience, Tokyo, Japan) and counterstained with hematoxylin, and then dehydrated and mounted onto cover slips.

To estimate the immunohistochemical expression of the CaSR and VDR in parathyroid glands, image analysis was performed using ImageScope (Aperio Technologies, Inc., Vista, CA, USA). Pathological images from all sections were captured in tiff format at 200× magnification. These images were analyzed using the Positive Pixel Count Algorithm v9. This algorithm quantifies the amount of staining in a specific area, and counts the number and intensity-sum in three intensity ranges (weak positive, positive, strong positive). Staining intensity was classified as negative (0), weak positive (1), positive (2), and strong positive (3) in each pixel. The intensity of immunohistochemical expression of the CaSR and VDR was assessed in each mouse.

### BrdU incorporation analyses

To identify the proliferating cells, BrdU-incorporated cells were detected using a BrdU immunohistochemistry kit (cat# ab125306, abcam plc., Cambridge,UK). All pathological images were scanned at 20 × magnification using the Aperio CS2 slide scanner (Leica biosystems). To identify proliferating cells, BrdU-positive cells were detected using HALO AI image analysis software (ver.3.2, indica labs). The regions of parathyroid glands in the entire image were specified using the annotation tool. The machine learning nuclei segmentation classifier algorithm was trained to recognize and segregate nuclei using shape and color. The proportion of BrdU-positive nuclei to the total number of nuclei in the entire parathyroid glands was calculated.

### Statistical analyses

Intergroup comparisons were made using the Tukey–Kramer method. Time-course changes in parameters were analyzed by Tukey’s Honest Significant Difference test. All data are presented as the mean ± SD. *P* < 0.05 was taken to indicate statistical significance. All statistical analyses were performed with commercially available software for Windows (JMP software version 12.0.1 (SAS Institute, Cary, NC)).

## Results

### Effects of a single administration of evocalcet on serum calcium and intact PTH levels in WT mice

**Fig. 1:**
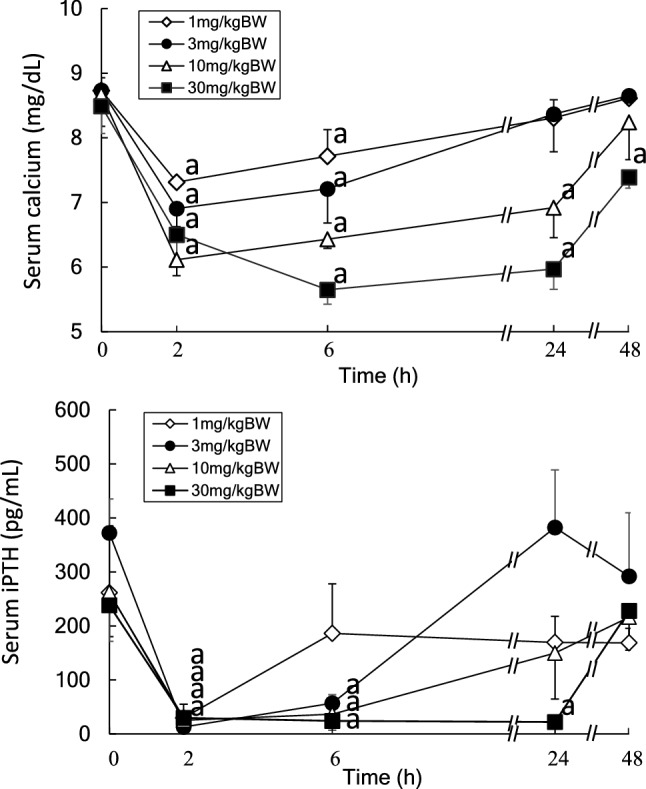
Effects of a single administration of evocalcet on serum calcium and intact PTH levels in wild-type (WT) mice (*N* = 5). Ca, calcium; iPTH, intact parathyroid hormone. Values represent the mean ± S.D. Time-course changes in serum calcium and serum PTH were significant across all test substance dose groups, as determined by repeated measures ANOVA. ^a^*P* < 0.05 versus pre-administration b by Tukey’s Honest Significant Difference test

A single administration of evocalcet significantly suppressed the serum calcium levels at 2 h and 6 h (1, 3, 10, and 30 mg/kg BW). Higher doses of evocalcet prolonged the suppressed serum calcium levels until 24 h (10 and 30 mg/kg BW) or 48 h (30 mg/kg BW) (Fig. 1). A single administration of evocalcet also significantly suppressed the serum intact PTH levels at 2 h (1, 3, 10, and 30 mg/kg BW) and 6 h (3, 10, and 30 mg/kg BW). Higher doses of evocalcet prolonged the suppression of serum intact PTH levels until 24 h (30 mg/kg BW).

### Changes in the PTH–calcium setpoint, and maximum and minimum PTH secretion by evocalcet (0.025 mg/g in chow) or vehicle in PC mice

**Fig. 2:**
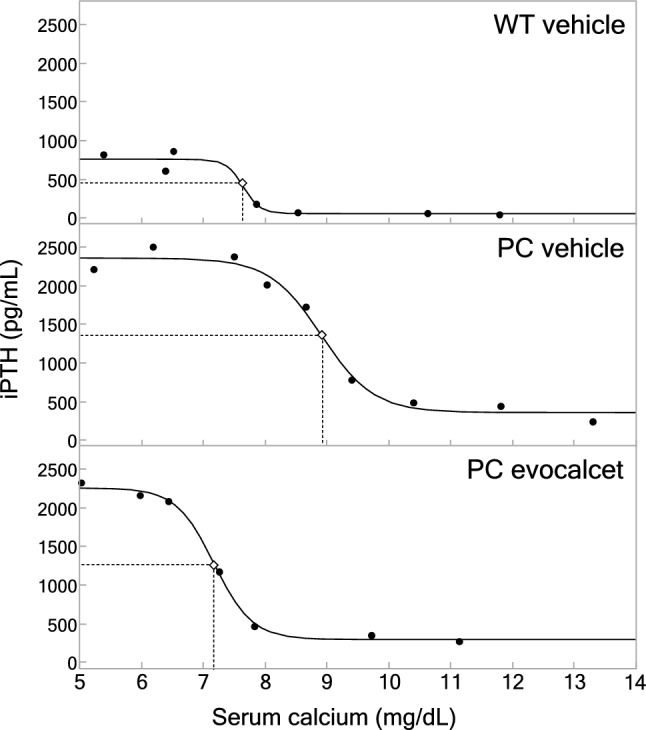
Example of an in vivo PTH–calcium sigmoidal curve and setpoint (indicated by the open diamond) in a WT mouse, a vehicle-treated PC mouse, and an evocalcet (0.025 mg/g in chow-treated PC mouse). Initially, a serum calcium concentration of below 7.0 mg/dL was not achieved in vehicle-treated PC mice, so for setpoint analysis, an additional 600 µmol/kg BW EGTA was administered

An example of an in vivo PTH–calcium sigmoidal curve and setpoint in a WT mouse, a vehicle-treated PC mouse, and an evocalcet-treated PC mouse was shown (Fig. 2). Nonlinear mixed-effects models were used to fit the sigmoidal curve [24]. A vehicle-treated PC mouse increased its setpoint and maximum PTH secretion compared to the vehicle-treated WT; otherwise, an evocalcet-treated PC mouse restored its setpoint to the vehicle-treated WT level, but its hill slope and maximum PTH secretion were not altered (Table 2). Goodness-of-fit statistics of setpoint analyses in individual mice were also shown in Table 3.

**Table 2:** Setpoint analyses in PC and WT mice

Subjects	WT vehicle (*N* = 8)	PC vehicle (*N* = 8)	PC evocalcet (*N* = 8)
Setpoint (mg/dL)	7.91 [7.47, 8.34]	8.71 [8.48, 8.95]^a^	7.02 [6.23, 7.80]^a,b^
Hill slope	− 16.09 [− 47.32, 15.15]	− 4.05 [− 6.97, − 1.13]	− 3.35 [− 5.22, − 1.47]
Maximum iPTH secretion (pg/mL)	779 [658, 900]	2338 [1177, 3498]^a^	2548 [837, 4259]^a^
Minimum iPTH secretion (pg/mL)	22 [5, 40]	211 [105, 316]	205 [3, 407]

Data are presented as mean and 95% confidence interval

^a^*P* < 0.05 *versus* WT vehicle, ^b^*P* < 0.05 *versus* PC vehicle by Tukey–Kramer

**Table 3:** Goodness-of-fit statistics of setpoint analyses in individual mice

Group	Mouse ID	AICc	BIC	SSE	MSE	RMSE	*R*^2^
WT vehicle	266	150	90	37,522	12,507	112	0.955
	261	153	92	54,551	18,184	135	0.952
	263	151	91	44,380	14,793	122	0.954
	124	123	93	14,308	3577	60	0.979
	908	144	84	17,128	5709	76	0.948
	847	147	87	24,167	8056	90	0.962
	913	147	86	22,859	7620	87	0.968
	948	140	80	9387	3129	56	0.989
PC vehicle	268	158	145	357,039	59,506	244	0.965
	865	139	120	100,205	20,041	142	0.985
	869	140	121	110,248	22,050	148	0.965
	911	128	109	27,856	5571	75	0.982
	918	152	133	388,233	77,647	279	0.991
	336	157	138	699,961	139,992	374	0.905
	345	131	112	40,759	8152	90	0.874
	367	145	115	222,150	55,537	236	0.968
PC evocalcet	868	143	83	13,847	4616	68	0.997
	864	164	104	274,256	91,419	302	0.966
	No tag	147	87	25,762	8587	93	0.962
	917	170	151	2,941,386	588,277	767	0.947
	338	124	105	17,314	3463	59	0.986
	343	168	108	522,686	174,229	417	0.918
	949L	108	89	3234	647	25	0.997
	950	157	97	99,617	33,206	182	0.964

*AICc* corrected Akaike information criterion; *BIC* Bayesian information criterion; *SSE* sum of squared errors; *MSE* mean squared error; *RMSE* root mean squared error; *R*^2^ coefficient of determination

### Effects of evocalcet on the proliferation of parathyroid cells

After 10 days administration of evocalcet or cinacalcet, serum iPTH and calcium levels were significantly suppressed compared to vehicle-treated PC mice, and significantly increased serum phosphate (Table 4). Serum creatinine, body weight, and food intake were not different among these groups.

**Table 4:** Serum parameters, body weight, and food intake at euthanasia and food intake during the experimental period in PC and WT mice

Subjects	WT vehicle (*N* = 10)	PC vehicle (*N* = 10)	PC cinacalcet (*N* = 10)	PC evocalcet (*N* = 10)
Serum iPTH (pg/mL)	220 ± 66	278 ± 82	133 ± 61^b^	138 ± 85^b^
Serum calcium (mg/dL)	8.5 ± 0.5	10.2 ± 0.7^a^	8.7 ± 0.7^b^	8.3 ± 0.6^b^
Serum phosphate (mg/dL)	7.2 ± 0.4	6.6 ± 0.5	7.7 ± 1.4^b^	7.9 ± 0.7^b^
Serum creatinine (mg/dL)	0.85 ± 0.05	0.87 ± 0.13	0.87 ± 0.07	0.87 ± 0.14
Body weight (g)	30.8 ± 4.4	30.9 ± 6.2	29.9 ± 6.2	32.1 ± 6.3
Food intake (g/day/100gBW)	15.4 ± 2.2	15.1 ± 2.6	13.8 ± 3.0	13.8 ± 2.9
Received dose (mg/day/100gBW)	0	0	13.8 ± 3.0	0.34 ± 0.07

Data are presented as mean ± SD

^a^*P* < 0.05 *versus* WT vehicle, ^b^*P* < 0.05 *versus* PC vehicle by Tukey–Kramer

**Fig. 3:**
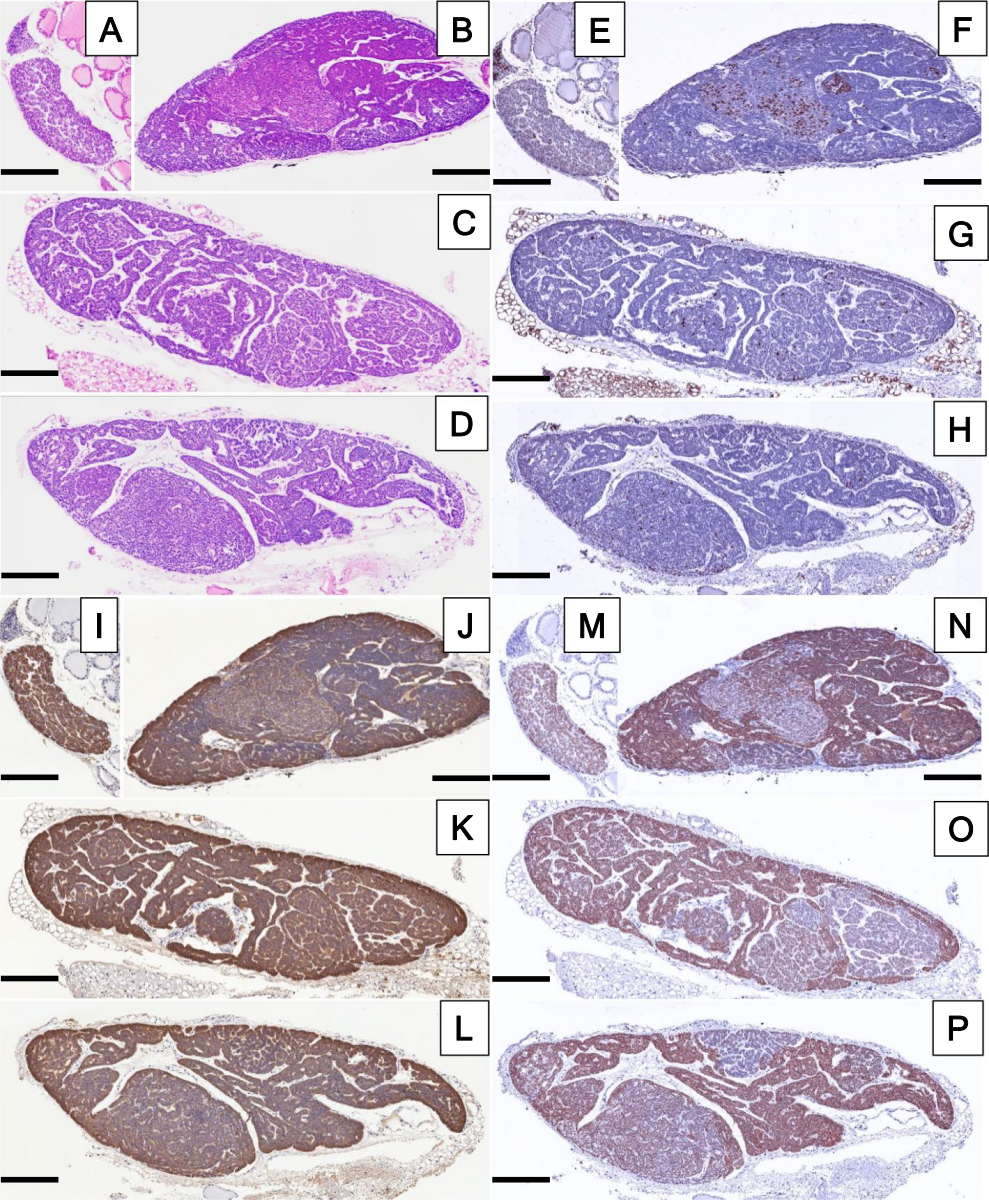
Immunohistochemical analyses of BrdU incorporation, CaSR and VDR expression in parathyroid glands. Parathyroid glands obtained from WT (**A**, **E**, **I**, **M**), PC vehicle-treated (**B**, **F**, **J**, **N**), PC cinacalcet-treated (**C**, **G**, **K**, **O**), and PC evocalcet-treated (**D**, **H**, **L**, **P**) mice. Parathyroid glands were stained with hematoxylin–eosin stain (**A**, **B**, **C**, **D**), an anti-BrdU antibody (**E**, **F**, **G**, **H**), an anti-CaSR antibody (**I**, **J**, **K**, **L**), and an anti-VDR antibody (**M**, **N**, **O**, **P**). BrdU uptake was strongly suppressed by cinacalcet (G) or evocalcet (**H**) in these glands. Magnification is 200x

**Fig. 4:**
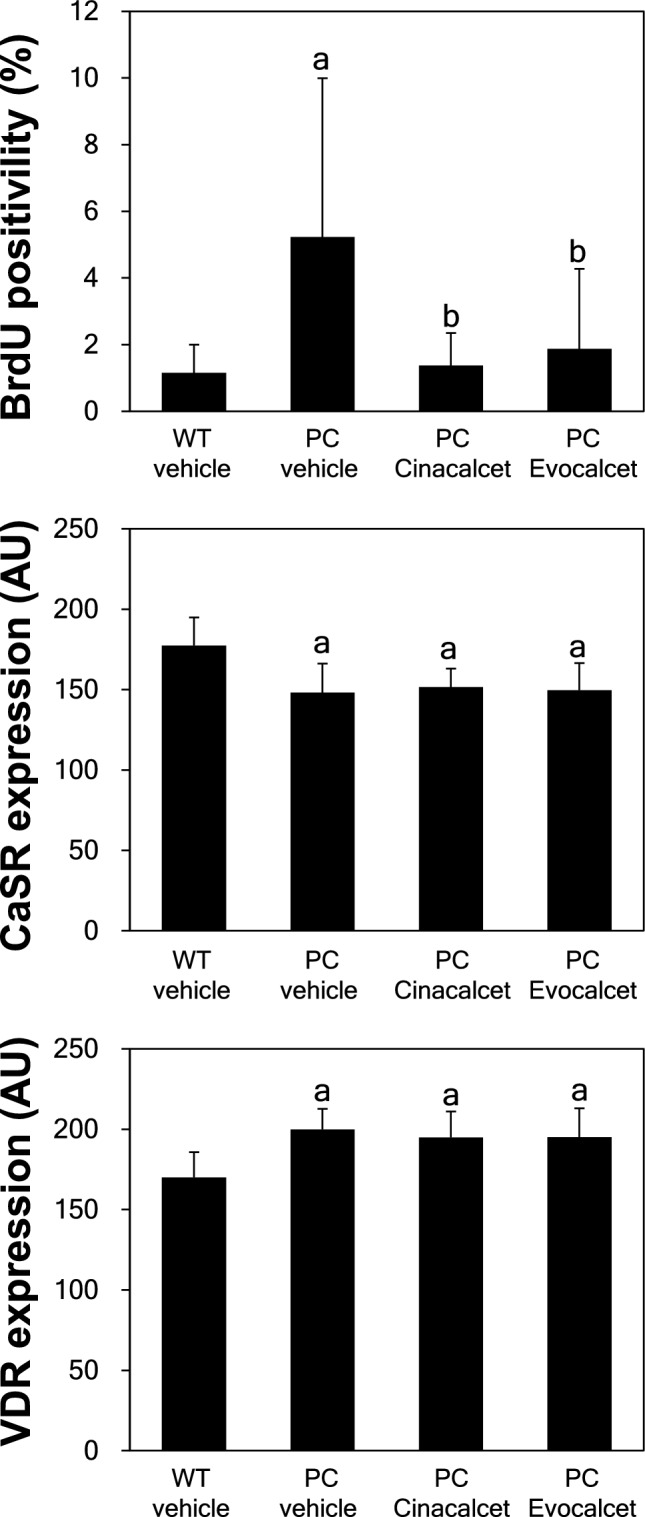
Intensity of immunohistochemical expression of BrdU incorporation, CaSR, and VDR expression in parathyroid glands (*N* = 20 glands per each group). AU; arbitrary unit. Data are presented as mean ± SD. Data are presented as mean ± SD. ^a^*P* < 0.05 vs WT vehicle-treated, ^b^*P* < 0.05 vs PC vehicle-treated by the Tukey–Kramer test

Examples of immunohistochemical analyses of BrdU incorporation, CaSR and VDR expression in parathyroid glands are shown (Fig. 3). The parathyroid cell proliferation rate showed a significant increase in PC mice compared to WT mice, as measured by BrdU uptake (Fig. 4). A significant reduction in BrdU uptake, similar level to that observed in WT mice, was seen in PC mice following the dietary administration of cinacalcet or evocalcet compared to vehicle-treated PC mice. Parathyroid CaSR expression in vehicle-treated PC mice was significantly reduced compared to WT mice, and no significant changes were observed after treatment with cinacalcet or evocalcet (Fig. 4). Parathyroid VDR expression in vehicle-treated PC mice was significantly increased compared to WT mice, and no significant changes were observed with cinacalcet or evocalcet treatment (Fig. 4).

## Discussion

This study highlights the therapeutic potential of evocalcet as a promising agent for the management of hyperparathyroidism. By effectively modulating the PTH–Ca setpoint and inhibiting parathyroid cell proliferation, evocalcet demonstrates characteristics consistent with those of established calcimimetics such as cinacalcet. These findings suggest that evocalcet may not only alleviate hyperparathyroidism symptoms by reducing PTH secretion, but also potentially impede disease progression by targeting parathyroid cell proliferation.

Uremic rats with SHPT due to 5/6-nephrectomy or adenine administration are useful animal models for CKD and have been used for the development of calcimimetics; however, these models have limitations, such as a short lifespan, and display a less severe form of SHPT that is distinct from SHPT in CKD patients receiving maintenance hemodialysis [26]. PC mice, with parathyroid-targeted overexpression of the *cyclin D1* oncogene, not only develop abnormal parathyroid cell proliferation, but also develop biochemical hyperparathyroidism with an abnormal PTH–calcium relationship. These features enable more clinically relevant testing of drugs such as cinacalcet [23, 25].

Data from single administrations of evocalcet indicated that a dosage of 0.025 mg/g in chow was suitable for subsequent experiments, including PTH–calcium setpoint analyses and parathyroid cell proliferation studies, ensuring consistent suppression of biochemical PHPT. The dosage of cinacalcet at 1 mg/g in chow was chosen as previously described [23]. In this study, the setpoint in PC mice treated with vehicle was significantly higher than the setpoint in WT mice, which is consistent with a previous study [21]. This is also consistent with the previously described in vitro study in primary cultured parathyroid cells obtained from PHPT and SHPT patients [27, 28]. The increase in the PTH–calcium setpoint is associated with a reduction in CaSR expression in the parathyroid, which is linked to these hyperfunctioning parathyroid diseases [21].

Evocalcet and cinacalcet both bind the same amino acid Glu837 of the 7-transmembrane regions of CaSR, [17] and have demonstrated positive allosteric effects on CaSR in vitro [18, 20]. However, the PTH–calcium setpoint, a key marker of positive allosteric effects, has not been evaluated in vivo in 5/6-nephrectomized rats. In this study, evocalcet significantly reduced the PTH–calcium setpoint without changing the maximum and minimum PTH levels in PC mice, indicating that evocalcet acted in the same way as calcimimetics such as cinacalcet. These phenomena were also observed in the PC mice administered the calcimimetic etelcalcetide [22], which binds in the Venus fly trap (VFT) domain of the CaSR at a site distinct from the calcium ion-binding sites in the hinge region [29]. Etelcalcetide functions as both a positive allosteric modulator and an agonist of the CaSR [30]. The distinct binding sites of these calcimimetics on the CaSR may account for their differing mechanisms of action and functional outcomes.

Cinacalcet suppressed parathyroid cell proliferation, as well as PTH secretion, in 5/6-nephrectomized uremic rats [31]. The suppressive effects of cinacalcet on parathyroid cell proliferation were also shown in PC mice, in which parathyroid cell proliferation was accelerated by *cyclin D1* oncogene overexpression [23]. In this study, evocalcet suppressed the parathyroid cell proliferation in PC mice as effectively as cinacalcet. Since cyclin D1 overexpression is observed in parathyroid tumors associated with PHPT [32], these findings suggest that evocalcet may potentially slow disease progression by targeting parathyroid cell proliferation. However, this effect was not observed in patients with PHPT. Conversely, a long-term study in hemodialysis patients with SHPT demonstrated that evocalcet effectively suppressed the PTH secretion and maintained low PTH levels, which correlated with a reduction in parathyroid gland volume [33]. These results indicate that evocalcet may help inhibit the progression of SHPT.

A previous report [34] demonstrated that parathyroid CaSR downregulation occurs prior to biochemical abnormalities, whereas parathyroid VDR downregulation follows the onset of mild to severe PHPT. In this study, the mice exhibited mild PHPT, and their parathyroid CaSR expression was reduced compared to that of wild-type parathyroids. Conversely, parathyroid VDR expression was higher than in wild-type parathyroid glands due to increased cell density resulting from parathyroid hypercellularity. Although cinacalcet and evocalcet upregulate decreased CaSR and VDR expression levels in parathyroid glands in uremic rats [35, 36], cinacalcet failed to upregulate parathyroid CaSR and VDR expression in PC mice [23]. In this study, evocalcet also could not upregulate parathyroid CaSR and VDR expression in PC mice, which parathyroid tissue overexpresses *cyclin D1* oncogene. Although direct molecular relationships between the driving *cyclin D1* oncogene and parathyroid receptors such as CaSR and VDR are not established, a prospective cohort study showed that symptomatic PHPT patients had significantly greater *cyclin D1* mRNA expression and lower CaSR expression than asymptomatic patients [32]. Further examination is necessary to investigate the role of the parathyroid *cyclin D1* oncogene on parathyroid receptors. Immunohistochemistry was used for quantifying CaSR and VDR expression in parathyroid glands. While Western blot or qRT-PCR are recognized as more precise quantitative methods, mouse parathyroid tumors are extremely small, providing insufficient sample material for these techniques. Therefore, we employed immunohistochemistry, which has established precedent in previous reports [22, 23, 25].

A limitation of this study is that the in vivo experimental system, while advantageous for many reasons, is not suitable for directly detecting the allosteric activity of evocalcet as compared with in vitro experimental systems [18]. In this study, we used PC mice as a model of PHPT; however, parathyroid-targeted *cyclin D1* overexpression is not the sole cause of PHPT. The mice used in this study had mild hyperparathyroidism, and a single dose of evocalcet was used for PTH-calcium setpoint analyses and parathyroid proliferation analyses. Another limitation is the absence of data on bone, kidney stone, or cardiovascular outcomes, which could not be evaluated in this study. A high dose of cinacalcet suppressed PTH secretion in PC mice with severe hyperparathyroidism [25]; however, in our study, moderate doses of evocalcet administered to PC mice with mild hyperparathyroidism suppressed the PTH secretion. Further studies are required to examine the potency of a high dose of evocalcet in PC mice with severe hyperparathyroidism. Another previously described limitation [24] of in vivo PTH–calcium setpoint analyses is that animals cannot achieve the same low and/or high serum calcium concentrations as primary cultured parathyroid cells in vitro. In addition, a mouse model of severe hyperparathyroidism is not suitable for setpoint analysis, since it is difficult to use severe hyperparathyroidism in mice to obtain minimum PTH secretion, and mice sometimes die of hypercalcemia when calcium gluconate administration is used for setpoint analysis. For this reason, mice with mild hyperparathyroidism were utilized in this study.

In conclusion, our study highlights the therapeutic potential of evocalcet, which has improved GI toxicity over cinacalcet, as a promising agent for the management of hyperparathyroidism. By effectively modulating the setpoint and inhibiting parathyroid cell proliferation, evocalcet demonstrates characteristics consistent with those of established calcimimetics such as cinacalcet. These findings suggest that evocalcet may not only alleviate the consequences of hyperparathyroidism by reducing the PTH secretion, but may also potentially impede disease progression by targeting the parathyroid cell proliferation.

## References

[1] Imanishi Y, Inaba M, Kawata T, Nishizawa Y (2009) Cinacalcet in hyperfunctioning parathyroid diseases. Ther Apher Dial 13:S7–S11 19765257 10.1111/j.1744-9987.2009.00768.x

[2] Malberti F, Farina M, Imbasciati E (1999) The pth-calcium curve and the set point of calcium in primary and secondary hyperparathyroidism. Nephrol Dial Transpl 14:2398–2406 10.1093/ndt/14.10.2398 10528664

[3] Brown EM (1983) Four-parameter model of the sigmoidal relationship between parathyroid hormone release and extracellular calcium concentration in normal and abnormal parathyroid tissue. J Clin Endocrinol Metab 56:572–581 6822654 10.1210/jcem-56-3-572

[4] Kifor O, Moore FD Jr., Wang P, Goldstein M, Vassilev P, Kifor I, Hebert SC, Brown EM (1996) Reduced immunostaining for the extracellular Ca^2+^-sensing receptor in primary and uremic secondary hyperparathyroidism. J Clin Endocrinol Metab 81:1598–1606 8636374 10.1210/jcem.81.4.8636374

[5] Nemeth EF, Steffey ME, Hammerland LG, Hung BC, Van Wagenen BC, DelMar EG, Balandrin MF (1998) Calcimimetics with potent and selective activity on the parathyroid calcium receptor. Proc Natl Acad Sci U S A 95:4040–4045 9520489 10.1073/pnas.95.7.4040 PMC19959

[6] Moe S, Drueke T, Cunningham J, Goodman W, Martin K, Olgaard K, Ott S, Sprague S, Lameire N, Eknoyan G, Kidney Disease: Improving Global O, (2006) Definition, evaluation, and classification of renal osteodystrophy: a position statement from Kidney Disease: Improving Global Outcomes (KDIGO). Kidney Int 69:1945–1953 16641930 10.1038/sj.ki.5000414

[7] Moe SM, Cunningham J, Bommer J, Adler S, Rosansky SJ, Urena-Torres P, Albizem MB, Guo MD, Zani VJ, Goodman WG, Sprague SM (2005) Long-term treatment of secondary hyperparathyroidism with the calcimimetic cinacalcet HCl. Nephrol Dial Transpl 20:2186–2193 10.1093/ndt/gfh966 16030053

[8] Block GA, Bushinsky DA, Cunningham J, Drueke TB, Ketteler M, Kewalramani R, Martin KJ, Mix TC, Moe SM, Patel UD, Silver J, Spiegel DM, Sterling L, Walsh L, Chertow GM (2017) Effect of Etelcalcetide vs placebo on serum parathyroid hormone in patients receiving hemodialysis with secondary hyperparathyroidism: two randomized clinical trials. JAMA 317:146–155 28097355 10.1001/jama.2016.19456

[9] Fukagawa M, Shimazaki R, Akizawa T, Evocalcet study g, (2018) Head-to-head comparison of the new calcimimetic agent evocalcet with cinacalcet in Japanese hemodialysis patients with secondary hyperparathyroidism. Kidney Int 94:818–825 30049473 10.1016/j.kint.2018.05.013

[10] Shigematsu T, Koiwa F, Isaka Y, Fukagawa M, Hagita K, Watanabe YS, Honda D, Akizawa T (2023) Efficacy and safety of Upacicalcet in hemodialysis patients with secondary hyperparathyroidism: a randomized placebo-controlled trial. Clin J Am Soc Nephrol 18:1300–1309 37696667 10.2215/CJN.0000000000000253 PMC10578632

[11] Bilezikian JP, Bandeira L, Khan A, Cusano NE (2018) Hyperparathyroidism. Lancet 391:168–178 28923463 10.1016/S0140-6736(17)31430-7

[12] Marcocci C, Chanson P, Shoback D, Bilezikian J, Fernandez-Cruz L, Orgiazzi J, Henzen C, Cheng S, Sterling LR, Lu J, Peacock M (2009) Cinacalcet reduces serum calcium concentrations in patients with intractable primary hyperparathyroidism. J Clin Endocrinol Metab 94:2766–2772 19470620 10.1210/jc.2008-2640 PMC3214593

[13] Takeuchi Y, Takahashi S, Miura D, Katagiri M, Nakashima N, Ohishi H, Shimazaki R, Tominaga Y (2017) Cinacalcet hydrochloride relieves hypercalcemia in Japanese patients with parathyroid cancer and intractable primary hyperparathyroidism. J Bone Miner Metab 35:616–622 27873072 10.1007/s00774-016-0797-0

[14] Takeuchi Y, Nishida Y, Kondo Y, Imanishi Y, Fukumoto S (2020) Evocalcet in patients with primary hyperparathyroidism: an open-label, single-arm, multicenter, 52-week, dose-titration phase III study. J Bone Miner Metab 38:687–694 32274572 10.1007/s00774-020-01097-y

[15] Block GA, Martin KJ, de Francisco AL, Turner SA, Avram MM et al (2004) Cinacalcet for secondary hyperparathyroidism in patients receiving hemodialysis. N Engl J Med 350:1516–1525 15071126 10.1056/NEJMoa031633

[16] Gincherman Y, Moloney K, McKee C, Coyne DW (2010) Assessment of adherence to cinacalcet by prescription refill rates in hemodialysis patients. Hemodial Int 14:68–72 19732171 10.1111/j.1542-4758.2009.00397.x

[17] Miyazaki H, Ikeda Y, Sakurai O, Miyake T, Tsubota R et al (2018) Discovery of evocalcet, a next-generation calcium-sensing receptor agonist for the treatment of hyperparathyroidism. Bioorg Med Chem Lett 28:2055–2060 29724589 10.1016/j.bmcl.2018.04.055

[18] Kawata T, Tokunaga S, Murai M, Masuda N, Haruyama W, Shoukei Y, Hisada Y, Yanagida T, Miyazaki H, Wada M, Akizawa T, Fukagawa M (2018) A novel calcimimetic agent, evocalcet (MT-4580/KHK7580), suppresses the parathyroid cell function with little effect on the gastrointestinal tract or CYP isozymes in vivo and in vitro. PLoS ONE 13:e0195316 29614098 10.1371/journal.pone.0195316 PMC5882164

[19] Tokunaga S, Kawata T (2021) The effect of evocalcet on vagus nerve activity of the gastrointestinal tract in miniature pigs. PLoS ONE 16:e0245785 33481922 10.1371/journal.pone.0245785 PMC7822337

[20] Liu F, Wu CG, Tu CL, Glenn I, Meyerowitz J, Kaplan AL, Lyu J, Cheng Z, Tarkhanova OO, Moroz YS, Irwin JJ, Chang W, Shoichet BK, Skiniotis G (2024) Large library docking identifies positive allosteric modulators of the calcium-sensing receptor. Science 385:eado1868 39298584 10.1126/science.ado1868 PMC11629082

[21] Imanishi Y, Hosokawa Y, Yoshimoto K, Schipani E, Mallya S, Papanikolaou A, Kifor O, Tokura T, Sablosky M, Ledgard F, Gronowicz G, Wang TC, Schmidt EV, Hall C, Brown EM, Bronson R, Arnold A (2001) Primary hyperparathyroidism caused by parathyroid-targeted overexpression of cyclin D1 in transgenic mice. J Clin Invest 107:1093–1102 11342573 10.1172/JCI10523 PMC209274

[22] Hayashi N, Imanishi Y, Hirakawa T, Kobayashi I, Tateishi T, Miyaoka D, Nagata Y, Mori K, Morioka T, Inoue A, Harada K, Inaba M, Emoto M (2021) Etelcalcetide decreases the PTH-calcium setpoint without changing maximum and minimum PTH secretion in mice with primary hyperparathyroidism. J Bone Miner Metab 39:430–438 33196900 10.1007/s00774-020-01169-z

[23] Imanishi Y, Kawata T, Kenko T, Wada M, Nagano N, Miki T, Arnold A, Inaba M (2011) Cinacalcet HCl suppresses Cyclin D1 oncogene-derived parathyroid cell proliferation in a murine model for primary hyperparathyroidism. Calcif Tissue Int 89:29–35 21541686 10.1007/s00223-011-9490-4

[24] Imanishi Y, Hall C, Sablosky M, Brown EM, Arnold A (2002) A new method for in vivo analysis of parathyroid hormone-calcium set point in mice. J Bone Miner Res 17:1656–1661 12211436 10.1359/jbmr.2002.17.9.1656

[25] Kawata T, Imanishi Y, Kobayashi K, Kenko T, Wada M, Ishimura E, Miki T, Nagano N, Inaba M, Arnold A, Nishizawa Y (2005) Relationship between parathyroid calcium-sensing receptor expression and potency of the calcimimetic, cinacalcet, in suppressing parathyroid hormone secretion in an *in vivo* murine model of primary hyperparathyroidism. Eur J Endocrinol 153:587–594 16189180 10.1530/eje.1.02007

[26] Imanishi Y, Inaba M, Kawata T, Nishizawa Y (2009) Animal models of hyperfunctioning parathyroid diseases for drug development. Expert Opin Drug Discov 4:727–740 23489166 10.1517/17460440903022743

[27] Brown EM, Gardner DG, Brennan MF, Marx SJ, Spiegel AM, Attie MF, Downs RW Jr., Doppman JL, Aurbach CD (1979) Calcium-regulated parathyroid hormone release in primary hyperparathyroidism: studies in vitro with dispersed parathyroid cells. Am J Med 66:923–931 453225 10.1016/0002-9343(79)90446-7

[28] Brown EM, Wilson RE, Eastman RC, Pallotta J, Marynick SP (1982) Abnormal regulation of parathyroid hormone release by calcium in secondary hyperparathyroidism due to chronic renal failure. J Clin Endocrinol Metab 54:172–179 7054214 10.1210/jcem-54-1-172

[29] Mun HC, Franks AH, Culverston EL, Krapcho K, Nemeth EF, Conigrave AD (2004) The Venus fly trap domain of the extracellular Ca2+ -sensing receptor is required for L-amino acid sensing. J Biol Chem 279:51739–51744 15579475 10.1074/jbc.M406164/200

[30] Walter S, Baruch A, Dong J, Tomlinson JE, Alexander ST, Janes J, Hunter T, Yin Q, Maclean D, Bell G, Mendel DB, Johnson RM, Karim F (2013) Pharmacology of AMG 416 (Velcalcetide), a novel peptide agonist of the calcium-sensing receptor, for the treatment of secondary hyperparathyroidism in hemodialysis patients. J Pharmacol Exp Ther 346:229–240 23674604 10.1124/jpet.113.204834

[31] Colloton M, Shatzen E, Miller G, Stehman-Breen C, Wada M, Lacey D, Martin D (2005) Cinacalcet HCl attenuates parathyroid hyperplasia in a rat model of secondary hyperparathyroidism. Kidney Int 67:467–476 15673294 10.1111/j.1523-1755.2005.67103.x

[32] Kaur P, Hegde D, Singh P, Gautam D, Sarin D, Bhadada S, Mithal A (2024) mRNA expression of vitamin D receptor, calcium-sensing receptor, cyclin D1, and PTH in symptomatic and asymptomatic primary hyperparathyroidism. Eur J Endocrinol 191:457–462 39353070 10.1093/ejendo/lvae122

[33] Nagano N, Ishikawa T, Yamaguchi M, Katsuragi Y, Miya M, Tamei N, Muto S, Tsutsui T, Ogawa T, Ito K (2024) Long‑term treatment of evocalcet in hemodialysis patients with secondary hyperparathyroidism: a five‑year prospective cohort study in 147 Japanese patients. Renal Replace Ther 10:13

[34] Mallya SM, Gallagher JJ, Wild YK, Kifor O, Costa-Guda J, Saucier K, Brown EM, Arnold A (2005) Abnormal parathyroid cell proliferation precedes biochemical abnormalities in a mouse model of primary hyperparathyroidism. Mol Endocrinol 19:2603–2609 15928311 10.1210/me.2005-0116

[35] Mizobuchi M, Hatamura I, Ogata H, Saji F, Uda S, Shiizaki K, Sakaguchi T, Negi S, Kinugasa E, Koshikawa S, Akizawa T (2004) Calcimimetic compound upregulates decreased calcium-sensing receptor expression level in parathyroid glands of rats with chronic renal insufficiency. J Am Soc Nephrol 15:2579–2587 15466262 10.1097/01.ASN.0000141016.20133.33

[36] Saito T, Mizobuchi M, Sakai M, Kawata T, Kitayama T, Kato T, Suzuki T, Ogata H, Koiwa F, Honda H (2023) Effects of evocalcet on parathyroid calcium-sensing receptor and vitamin D receptor expression in uremic rats. FASEB J 37:e23094 37462513 10.1096/fj.202300209R

